# An Overview of Systematic Reviews of Shenmai Injection for Healthcare

**DOI:** 10.1155/2014/840650

**Published:** 2014-02-12

**Authors:** Ling-yan Lu, Guo-qing Zheng, Yan Wang

**Affiliations:** ^1^Department of Cardiology, The Second Affiliated Hospital of Wenzhou Medical University, Wenzhou 325027, China; ^2^Department of Neurology, The Second Affiliated Hospital of Wenzhou Medical University, Wenzhou 325027, China

## Abstract

Shenmai injection (SMI) is widely applied in clinical practice as an organ protector. This overview is to evaluate the current evidence from systematic reviews (SRs) of SMI for healthcare. The literature searches were carried out in 6 databases without language restrictions until December 2012. The quality of the primary studies from the respective SRs was evaluated by using Jadad score. The overview quality assessment questionnaire (OQAQ) was used to evaluate the methodological quality of all included SRs. Twenty eligible SRs were identified. They reported a wide range of conditions, including SMI for cardio/cerebrovascular diseases, viral myocarditis, tumor chemotherapy, and adverse drug reactions. Most of the primary studies were of good quality only in 1 SR of non-small-cell lung cancer. According to the OQAQ scores, the quality of included SRs was variable and six reviews were of high quality with a score of 5 points. Two SRs showed that SMI had low adverse drug reaction occurrence. In conclusion, there is mixed evidence to support efficacy of SMI for an adjunct therapy to tumor chemotherapy and premature evidence for the use of SMI for cardio/cerebrovascular disorders and viral myocarditis. SMI seems generally safe for clinical application. Further large sample-size and well-designed RCTs are needed.

## 1. Introduction

Shenmai injection (SMI) is derived from the famous traditional Chinese herbal prescription Shendong yin, whose formulation was first recorded in Zhengyin Maizhi (*Pattern, Cause, Pulse, and Treatment*) by Jing-Ming Qin in 1702 AD [1]. Shendong Yin consists of ginseng (Radix Ginseng, the root of *Panax ginseng* C.A. Mey., Araliaceae) and dwarf lilyturf tuber (Radix Ophiopogonis, the tuber of *Ophiopogon japonicus* (Thunb.) Ker-Gawl., Liliaceae) [1]. In modern time, Shendong Yin is still widely used in clinical practice in many countries such as China, Korea, Japan, and Singapore [2, 3]. According to the theory of traditional Chinese medicine, Shendong Yin has the function of invigorating qi for relieving desertion, nourishing Yin, and replenishing bodily fluids [1, 4]. Ginsenosides and ophiopogonis, isolated from ginseng and dwarf lilyturf tuber, were regarded as the principal constituents responsible for the pharmacological activities [5]. Using Chinese herbal medicine as injections is an innovation that has been proved to be effective in extensive clinical use in Mainland China. SMI was developed as a traditional Chinese patent injection since 1995. Various pharmacological studies indicated that SMI has immunomodulatory effects against tissue damage by suppressing the release of tumor necrosis factor alpha (TNF-*α*), nitric oxide (NO), and other inflammatory products from macrophages, and it also has protective effects of dilating the coronary arteries, increasing blood supply, improving microcirculation, and eliminating surplus free radicals [6]. Because of its effectiveness, nowadays SMI has become a famous Chinese patent injection and has been widely applied in clinical practice as an organ protector [3, 6].

Evidence-based medicine (EBM) is a strategy for the critical evaluation and uniform comparison of clinical trial data with conclusions according to predetermined efficacy criteria [7]. Several systematic reviews have been conducted to evaluate SMI for a wide range of conditions in clinical practice, including viral myocarditis, acute myocardial infarction, acute cerebral infarction, tumor (as an adjunct therapy to chemotherapy), coronary heart disease, chronic cor pulmonale, dilated cardiomyopathy, and heart failure [8–25]. However, the results are all positive which basically come from the poor methodological quality. Thus, the objective of this overview is to critically summarize and evaluate systematic reviews of SMI for healthcare.

## 2. Methods

### 2.1. Eligibility Criteria

Systematic review is defined as an exhaustive review of the literature addressing a clearly defined question, which uses a systematic and explicit methodology to identify, select, and critically evaluate all the relevant studies and collect and analyse the data emerging from the studies included in it [26]. Systematic reviews must include an explicit and repeatable methods section describing the search strategy and explicit inclusion/exclusion criteria [27]. To be considered, the systematic review has to be concerned specifically with the effectiveness of SMI and must include evidence from at least two randomized controlled trials (RCTs). Systematic reviews evaluating SMI together with other Chinese herbal medicine (CHM) and without separate evaluation of the individual drug were excluded. Reviews, comments, and overviews without a systematic methods section were excluded.

### 2.2. Literature Search

**Figure 1:**
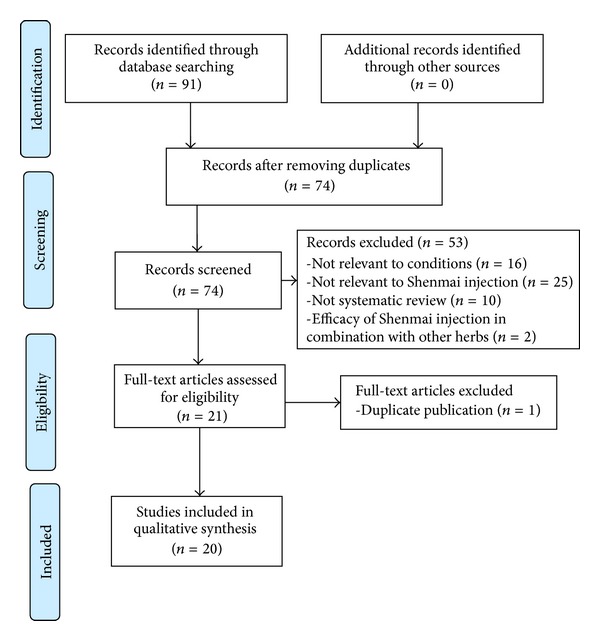
PRISMA 2009 Flow Diagram.

Electronic literature search was carried out in the following databases up to December 31, 2012 without language restrictions: Pubmed, EMBASE, Chinese Hospital Knowledge Database (CHKD), China National Knowledge Infrastructure, VIP Journals Database, and Wanfang Med Online Database (Figure 1). The keywords used in the search were “systematicreview OR meta-analysis” AND “Shenmai.” Chinese database was searched using the above search terms in Chinese accordingly.

### 2.3. Study Selection and Data Collection Process

Data were extracted and evaluated independently by 2 authors using predefined criteria. Disagreements were resolved through consultation with corresponding author. The assessments of the quality of the primary studies which were evaluated by using Jadad score were adopted from the systematic reviews, respectively.

### 2.4. The Overview Quality Assessment Questionnaire

The overview quality assessment questionnaire (OQAQ) [28–30] which consists of 10 questions was used to evaluate the methodological quality of all included systematic reviews. Questions 1 to 9, which were answered with “adequate,” “inadequate,” or “not mentioned,” addressed the 5 methodological aspects of systematic reviews including search strategy, study selection, validity assessment, data analysis, and inferences. The final question 10 is the overall scientific quality of the review article graded on a 7-point scale. A score of three or less was considered as indicative of extensive or major flaws and a score of 5 or more as suggesting minor or minimal flaws. Two authors assess the OQAQ independently. We settled any discrepancies by discussion or consulting with corresponding author.

## 3. Results

### 3.1. Description of the Screening Process

Our searches generated 91 potentially relevant articles. After removal of duplicates, 74 records remained. Through screening titles and abstracts, we excluded 53 papers with at least one of following reasons: (1) not relevant to conditions; (2) not relevant to SMI; (3) not systematic review; and (4) efficacy of SMI in combination with other CHM. After full-text evaluation on the remaining 21 articles, 1 article was excluded for duplicate publication [31]. Ultimately, 20 eligible studies were selected in present study. Of the 20 eligible studies, two systematic reviews were specifically focused on the adverse drug reactions (ADRs) of SMI [32, 33], which we would analysis in a separate part. The screening process is summarized in a flow diagram (Figure 1).

### 3.2. Study Characteristics

Two systematic reviews were published in English [24, 33] and 18 others in Chinese from 2005 to 2012. The first authors of all included trials were from China and were affiliated to academic institutions. They reported a wide range of conditions, including viral myocarditis (*n* = 4), acute myocardial infarction (*n* = 3), acute cerebral infarction (*n* = 3), tumor (SMI as an adjunct therapy to chemotherapy) (*n* = 2), coronary heart disease (*n* = 3), chronic cor pulmonale (*n* = 1), dilated cardiomyopathy (*n* = 1), and heart failure (*n* = 1). The systematic reviews were based on 6 to 33 primary studies. Most of the primary studies were of good quality only in 1 systematic review [17], while almost all the primary studies were of poor quality or C grade in the other 17 systematic reviews according to the Jadad score. All systematic reviews applied a meta-analytic approach. Key data of the included systematic reviews are summarized in Table 1.

### 3.3. Assessing the Quality of Systematic Reviews

According to the OQAQ scores, the quality of these reviews were varied. Six systematic reviews included were considered to having minor or minimal flaws. that is, scoring 5 points on the OQAQ [8, 9, 14, 17, 21, 24]. The other 12 systematic reviews were mostly of poor quality [10–13, 15, 16, 18–20, 22, 23, 25]. Among them, 3 systematic reviews scored 4 points [19, 22, 23], and the remaining 9 trials which had major flaws scored equal to or less than 3 points [10–13, 15, 16, 18, 20, 25]. The details of the assessment of the quality of systematic reviews are listed in Table 2.

### 3.4. Effectiveness

All of the systematic reviews reached positive conclusion (Table 1).

### 3.5. Adverse Drug Effects

There are 2 reviews addressing the adverse drug effects of SMI [32, 33]. One review was published in 2003 and only found 16 cases which were grouped into 4 main categories: skin lesions (*n* = 1), anaphylactic shock (*n* = 5), phlebitis (*n* = 1), cardiovascular and cerebrovascular diseases (*n* = 7), and other adverse effects (*n* = 2) [32]. The other review was carried out in 2010 in which they collected relevant information such as gender, age, allergic history, primary diseases treated in ADR cases, types, occurrence times, severity of ADRs, and menstruum and compatibility of SMI. Of the 822 total reported ADR cases, 246 ADR cases resulted from 181 ADR reports and 146 described a total of 576 ADR cases in 1828 clinical studies [33]. This systematic review found that the most commonly affected age group was 40 to 69. There are 36 cases which were described as having an allergic history. The diseases being treated in ADR cases were principally heart failure and coronary artery heart disease. Thirty-eight of the 246 ADR cases in ADR reports described anaphylactic shock, while the most common ADR reported in clinical studies was headache or dizziness. In classification of ADR severity for SMI, 38 cases, 48 cases, 99 cases, and 637 cases were class I, class II, class III, and class IV, respectively, and there was no death. All in all, this systematic review indicated that SMI had low ADR occurrence, although some potential factors such as irrational compatibility and dosages may lead to a high risk of ADR.

## 4. Discussions

### 4.1. Summary of Evidence

This overview indicated that a remarkable multitude of systematic reviews of SMI for many types of diseases has emerged between 2003 and 2012, suggesting that the interests of the public and the medical profession in the use of SMI for healthcare have grown considerably in recent years. SMI is commonly used in the cardiocerebrovascular area, in viral myocarditis, and in an adjunct therapy to tumor chemotherapy. There is mixed evidence to support efficacy of SMI for an adjunct therapy to tumor chemotherapy and premature evidence for the use of SMI for cardiocerebrovascular disorders and viral myocarditis, whereas SMI treatment was generally safe for healthcare.

### 4.2. Limitations

The OQAQ scale was selected to assess various aspects of the methodological quality of systematic reviews. However, only 6/18 scoring 5 or above indicates that the study has minimal flaws. Second, this review rest with inherent limitations in the primary studies. The only trial [17] is SMI as an adjunct therapy for patients with NSCLC based on more than half of the high quality of the included primary studies. Third, all the studies met the criteria coming from China was another weakness that potentially limited the generalizability of the findings. SMI belongs to Chinese traditional medicine patent injection and is only available in the domestic market. Thus, relevant researches are limited to Chinese researchers. Fourth, All of the systematic reviews reached positive conclusion. Several groups have shown that nearly 100% of all Chinese trials reported positive results [34]. Thus, the evidences have to be interpreted with caution. At last, due to the remarkable multitude of databases, the literature search may not be whole and some important data may be lost. This could have a potential influence on the outcomes.

### 4.3. Implication of Practice

This review revealed mixed evidence in support of the use of SMI for an adjunct therapy to tumor chemotherapy and premature evidence for the use of SMI for the cardiocerebrovascular area and viral myocarditis. Additionally, SMI appeared to be well tolerated in almost all included patients. However, it should be remembered that a lack of scientific evidence does not necessarily mean that the treatment is ineffective [35]. Since SMI is not available in the western countries, we suggested that oral administration of Shendong yin could be an alternative option for healthcare.

### 4.4. Cancer

There are two studies about SMI as an adjunct therapy for patients with cancer. One contains 13 primary studies which included in 1040 patients with all types of cancer [19] and the other contains 7 primary studies which included in 510 patients specifically with NSCLC [17]. As for the former, although it has rigorous and positive studies, the current evidence is insufficient for support of the effectiveness of SMI as an adjunct therapy of cancer due to the poor methodological quality in the primary studies. However, the evidence for NSCLC is supported by probable evidence for routinely clinical use because of its more than half high methodological quality of the included primary studies and scoring 5 points on the OQAQ. In pharmacological studies, the antitumor effects of a ginsenoside Rg3-fortified red ginseng preparation (Rg3-RGP) were investigated in human NSCLC (H460) cells using in vitro cytotoxicity assay and in vivo nude mouse xenograft model, suggesting that Rg3-RGP could exert antitumor activities through indirect immunomodulatory actions, without causing adverse effects as caused by doxorubicin [36].

### 4.5. Cardiocerebrovascular Diseases

Cardiocerebrovascular diseases are the leading cause of death worldwide and the first cause of acquired disability, and their costs both direct and indirect are astronomic. At least 12 SRs have been published in this area, suggesting that SMI is mainly and widely used in cardio/cerebrovascular diseases, including acute myocardial infarction (*n* = 3), acute cerebral infarction (*n* = 3), coronary heart disease (*n* = 3), chronic cor pulmonale (*n* = 1), dilated cardiomyopathy (*n* = 1), and heart failure (*n* = 1). Despite this wealth of clinical use, there are insufficient evidence to conclude efficacy of SMI for cardiocerebrovascular diseases because of low methodological quality of the primary included trials.

### 4.6. Viral Myocarditis

Viral myocarditis is one of the most challenging diseases to diagnose and treat in cardiology and has been commonly associated with a viral infection. Treatment of acute viral myocarditis is still an integrative and aggressive supportive care, except for giant cell myocarditis where immunotherapy has been shown to improve survival [37]. Currently, many patients turn to herbal medicine, a form of the main part of traditional Chinese medicine, when conventional medicine fails them or they believe strongly in the effectiveness of complementary medicine. An updated systematic review which was recently published by Liu et al. [38] indicated that numerous clinical trials have been conducted to investigate the efficacy and safety of herbal medicines for viral myocarditis, suggesting that there is no evidence of benefit of herbal medicine on all-cause mortality, and some herbal medicines may lead to improvement of ventricular premature beat, electrocardiogram, levels of myocardial enzymes, and cardiac function in viral myocarditis, alleviating myocardial damage, and improving the effect of treatment of viral myocarditis. In the present study, four SRs were selected for viral myocarditis. Only one SR is rigorous and based on a sufficiently large number of primary studies, which is scored 5 points. However, its primary studies are of poor methodological quality. Therefore, its positive conclusion should treated cautiously.

### 4.7. Implication of Research

A number of implications for research arise from this review. First, improvement in the methodological quality of primary RCTs is crucial for future clinical studies. We recommend that some specific guidelines such as the CONSORT 2010 statement [39] and guidelines for RCTs investigating CHM [40] and CONSORT for TCM [41] should be used as a combined guideline when designing and reporting RCTs for CHM. Second, SRs must be of high quality. The overall quality of present included SRs is not so good. Thus, there is room for further improvement of methodological quality of SRs. Reports of SRs and meta-analyses should use the PRISMA statement [42] as a guide and encourage the prospective registration of SRs. Third, the safety of herbal patent injection has become a major concern to both national health authorities and the general public. The quantity of published literatures ranked SMI the ninth in ADRs of 33 varieties of CHM Injections on the 2004 edition of National Essential Drugs List (2004 edition) of China [43]. In the present overview, SMI appears to be well tolerated. While reporting of ADR following CHM injection is becoming more and more common, the reporting quality is of concern. A standard reporting format for ADR has been developed [44] and we suggested that improvement of reporting of adverse events and ADRs of SMI may follow up. Fourth, SMI is widely used in the treatment of various disorders and seems generally safe. Although the current evidence is insufficient to support routine use of SMI, it is a promising candidate for further clinical trial of various disorders, especially an adjunct therapy to tumor chemotherapy, cardiocerebrovascular area, and viral myocarditis.

## 5. Conclusions

The interests of the public and the medical profession in the use of SMI for healthcare have grown considerably in recent years. There is mixed evidence to support efficacy of SMI for an adjunct therapy to tumor chemotherapy and premature evidence for the use of SMI for cardiocerebrovascular disorders and viral myocarditis. In addition, SMI seems generally safe for clinical application. Further large sample-size and well-designed RCTs are needed.

## Figures and Tables

**Table 1:** Study characteristics of included systematic reviews.

First author (year)	Condition	No. of primary studies	Quality of primary studies	Overall conclusion^+^	Meta-analysis	Quality of review (OQAQ)*	Result^+^ +/−
Zhang (2010) [9]	Viral myocarditis among children	15 (1018)	Poor	…may have some effect…	The total effective rate: SMI plus RT versus RT (6RCTs): RR 1.16, 95% CI (1.07, 1.25); SMI plus RT versus western medicine plus RT (5RCTs): RR 1.12, 95% CI (1.01, 1.25); SMI plus western medicine versus western medicine versus RT (4RCTs): RR 1.26, 95% CI (1.12, 1.42)	5	+

Li (2011) [24]	Viral myocarditis	10 (649)	Poor	…tend to be effective…	SMI plus routine therapy (RT) versus RT: The total effective rate: OR = 4.38, 95% CI (2.59, 7.38), *P* < 0.00001; The remarkable response rate: OR = 2.67, 95% CI (1.93, 3.71), *P* < 0.00001	3	+

Jin (2011) [10]	Viral myocarditis among children	7 (416)	Poor	…effective on…	SMI plus RT versus RT: RR = 1.25, 95% CI (1.15, 1.36), *P* = 0.000	2	+

Shi (2012) [16]	Viral myocarditis	12 (731)	Poor	…can increase the clinical efficacy…	SMI plus RT versus RT: The total effective rate: RR = 1.22, 95% CI (1.14, 1.30), *P* < 0.00001	1	+

Huang (2009) [23]	AMI	7 (901)	Poor (C grade)	…can decrease the mortality rate obviously…	SMI plus RT versus RT: the fatality rate during hospitalization: RR = 0.55, 95% CI (0.33, 0.90), *P* = 0.02	4	++

Zeng (2010) [21]	AMI	13 (1707)	Poor (C grade)	…can significantly decrease the mortality rate …	SMI plus RT versus RT: The mortality rate: RR = 0.55, 95% CI (0.39, 0.76), *P* = 0.0003	5	++

Hu (2012) [14]	AMI	15 (1806)	Poor	…can decrease the mortality rate …	SMI plus RT versus RT: the fatality rate during hospitalization: OR = 0.43, 95% CI (0.31, 0.60), *P* < 0.00001	5	+

Li (2006) [11]	Acute cerebral infarction	6 (477)	Poor	…have positive effects on…	SMI versus blank control: the total effective rate: RR = 1.20, 99% CI (1.09, 1.33), *P* < 0.00001	3	+

Shu (2008) [18]	Acute cerebral infarction	6 (563)	Mostly poor	…has a better effect on…	SMI versus compound salvia miltiorrhiza injection (CSMI): the total effective rate: OR = 4.73, 95% CI (2.61, 8.56), *P* < 0.01	2	++

Ma (2010) [12]	Acute cerebral infarction	9 (645)	Poor	…have positive effects on…	SMI plus RT versus RT: the total effective rate: RR = 1.21, 99% CI (1.10, 1.33), *P* < 0.00001	3	+

Liu (2005) [19]	An adjunct therapy to tumor chemotherapy	13 (1040)	Poor	…may have positive effects on…	SMI plus tumor chemotherapy versus tumor chemotherapy: the total effective rate: OR = 1.73, 95% CI (1.27, 2.34), *P* = 0.0004	4	+

Zhang (2010) [17]	An adjunct treatment of patients with NSCLC	7 (510)	Mostly good	…have positive effects on… (disease control rate and the occurrence of the gastrointestinal tract symptoms)	SMI plus tumor chemotherapy versus tumor chemotherapy: the total efficiency rate: OR = 1.45, 95% CI (1.01, 2.07), *P* < 0.05; the disease control rate: OR = 2.31, 95% CI (1.42, 3.73), *P* < 0.05; the occurrence of the gastrointestinal tract symptoms: OR = 0.37, 95% CI (0.26, 0.54), *P* < 0.05	5	+
Cui (2010) [22]	Coronary heart disease	7 (510)	Poor	…have positive effects on…	SMI plus RT versus RT Danshen injection plus RT: the total effective rate: OR = 6.71, 95% CI (2.53, 17.77), *P* = 0.001	4	+

Wang (2011) [15]	Coronary heart disease	21 (2219)	Mostly poor (4 B grade and 17 C grade)	…effective on… obviously	SMI plus RT versus RT: the total effective rate: OR = 3.43, 95% CI (2.73, 4.30), *P* < 0.01	3	++

Li (2012) [25]	Coronary heart disease	18 (1986)	Mostly Poor (2 articles 3 points and remaining 2 points)	…have positive effects on…	SMI plus RT versus RT: The total effective rate: RR = 1.31, 95% CI (1.20, 1.43), *P* < 0.0001; the ECG efficacy: RR = 1.46, 95% CI (1.32, 1.62), *P* < 0.0001; the improvement rate of NYHA: RR = 1.79, 95% CI (1.28, 2.51), *P* = 0.0006	2	+

Hou (2010) [8]	Heart Failure	15 (1174)	Mostly poor	…improve the therapeutic effect on…	SMI plus RT versus RT: the total effective rate: RR = 1.27, 95% CI (1.19, 1.35), *P* < 0.00001	5	+

Li (2011) [24]	Chronic pulmonary heart disease	33 (2617)	Poor	Potential effectiveness	SMI plus conventional medicine versus conventional medicine: the improvement in New York Heart Association classification of clinical status: OR = 0.24, 95% CI (0.19, 0.30), *P* < 0.00001	5	+

Liu (2012) [13]	Dilated cardiomyopathy	9 (688)	Poor (C grade)	…improve the therapeutic effect on…	SMI plus RT versus RT: the total effective rate: RR = 1.23, 95% CI (1.13, 1.33), *P* < 0.00001	3	+

Note: (OQAQ)^*∗*^ refers to the overall score of OQAQ which is from 1 to 7. OQAQ ≤ 3, extensive or major flaws; OQAQ ≥ 5, minor or minimal flaws. Conclusion +: As judged by the authors of the respective SRs. Result+: +, overall positive; −: fail to show effectiveness; ±, unclear.

**Table 2:** Overview quality assessment questionnaire (OQAQ) for the included systematic reviews.

First author (year) country	1 Were the search methods reported?*	2 Was the search comprehen-sive?*	3 Were the inclusion criteria reported?*	4 Was selection bias avoided?*	5 Were the validity criteria reported?**	6 Was validity assessed appropriately?**	7 Were the methods used to combine studies reported?**	8 Were the findings combined appropriately?**	9 Were the conclusions supported by the reported data?*	10 Overall score
Zhang (2010) [9]	Adequate	Adequate	adequate	Adequate	Not mentioned	Not mentioned	Adequate	Adequate	Adequate	5
Li (2011) [24]	Inadequate	Adequate	Adequate	Not mentioned	Not mentioned	Not mentioned	Adequate	Adequate	Adequate	3
Jin (2011) [10]	Inadequate	Inadequate	Adequate	Not mentioned	Not mentioned	Not mentioned	Adequate	Inadequate	Adequate	2
Shi (2012) [16]	Inadequate	Inadequate	Inadequate	Inadequate	Not mentioned	Not mentioned	Adequate	Adequate	Adequate	1
Huang (2009) [23]	Adequate	Inadequate	Adequate	Adequate	Not mentioned	Not mentioned	Adequate	Adequate	Adequate	4
Zeng (2010) [21]	Adequate	Adequate	Adequate	Adequate	Not mentioned	Not mentioned	Adequate	Adequate	Adequate	5
Hu (2012) [14]	Adequate	Adequate	Adequate	Adequate	Not mentioned	Not mentioned	Adequate	Adequate	Adequate	5
Li (2006) [11]	Inadequate	Adequate	Adequate	Not mentioned	Not mentioned	Not mentioned	Not mentioned	Adequate	Adequate	3
Shu (2008) [18]	Inadequate	Inadequate	Adequate	Not mentioned	Not mentioned	Not mentioned	Adequate	Adequate	Adequate	2
Ma (2010) [12]	Inadequate	Adequate	Adequate	Not mentioned	Not mentioned	Not mentioned	Adequate	Adequate	Adequate	3
Liu (2005) [19]	Adequate	Inadequate	Adequate	Adequate	Not mentioned	Not mentioned	Adequate	Adequate	Adequate	4
Zhang (2010) [17]	Adequate	Adequate	Adequate	Adequate	Not mentioned	Not mentioned	Adequate	Adequate	Adequate	5
Cui (2010) [22]	Inadequate	Adequate	Adequate	Adequate	Not mentioned	Not mentioned	Adequate	Adequate	Adequate	4
Wang (2011) [15]	Inadequate	Adequate	Adequate	Inadequate	Not mentioned	Not mentioned	Adequate	Adequate	Adequate	3
Li (2012) [25]	Inadequate	Inadequate	Adequate	Inadequate	Not mentioned	Not mentioned	Adequate	Adequate	Adequate	2
Hou (2010) [8]	Adequate	Adequate	Adequate	Adequate	Not mentioned	Not mentioned	Adequate	adequate	Adequate	5
Li (2011) [24]	Adequate	Adequate	Adequate	Adequate	Not mentioned	Not mentioned	Adequate	Adequate	Adequate	5
Liu (2012) [13]	Inadequate	Inadequate	Adequate	Adequate	Not mentioned	Not mentioned	Adequate	Adequate	Adequate	3

Note: *adequate: 1; inadequate and not mentioned: 0; **adequate: 0.5; inadequate and not mentioned: 0.
